# Infection with SARS-CoV-2 Variants Is Associated with Different Long COVID Phenotypes

**DOI:** 10.3390/v14112367

**Published:** 2022-10-27

**Authors:** Michele Spinicci, Lucia Graziani, Marta Tilli, Jerusalem Nkurunziza, Iacopo Vellere, Beatrice Borchi, Jessica Mencarini, Irene Campolmi, Leonardo Gori, Lorenzo Giovannoni, Carla Amato, Luca Livi, Laura Rasero, Francesco Fattirolli, Rossella Marcucci, Betti Giusti, Iacopo Olivotto, Sara Tomassetti, Federico Lavorini, Laura Maggi, Francesco Annunziato, Niccolò Marchionni, Lorenzo Zammarchi, Alessandro Bartoloni

**Affiliations:** 1 Department of Experimental & Clinical Medicine, University of Florence, 50134 Florence, Italy; 2 Infectious and Tropical Diseases Unit, Careggi University Hospital, 50134 Florence, Italy; 3 Department of Health Science, University of Florence, 50134 Florence, Italy; 4 Cardiac Rehabilitation Unit, Careggi University Hospital, 50134 Florence, Italy; 5 Atherothrombotic Disease Unit, Careggi University Hospital, 50134 Florence, Italy; 6 Cardiomyopathy Unit, Careggi University Hospital, 50134 Florence, Italy; 7 Department of Cardiothoracovascular Medicine, Careggi University Hospital, 50134 Florence, Italy; 8 Flow Cytometry Diagnostic Center and Immunotherapy (CDCI), Careggi University Hospital, 50134 Florence, Italy

**Keywords:** long COVID, post-COVID-19, variant, VOC, risk factors, predictors

## Abstract

COVID-19 has been associated with a broad range of long-term sequelae, commonly referred to as “long-COVID” or “post-COVID-19” syndrome. Despite an increasing body of literature, long COVID remains poorly characterized. We retrospectively analysed data from electronic medical records of patients admitted to the post-COVID-19 outpatient service of the Infectious and Tropical Diseases Unit, Careggi University Hospital, Florence, Italy, between June 2020 and June 2021, 4–12 weeks after hospital discharge. A total of 428 patients, 41% women, median age 64 years, underwent a follow-up visit a median 53 days after hospital discharge. Overall, 76% patients reported at least one persistent symptom, including dyspnoea (37%), chronic fatigue (36%), insomnia (16%), visual disorders (13%) and brain fog (13%). Increasing oxygen support (OR 1.4, 95% CI 1.1–1.8), use of immunosuppressants (OR 6.4, 95% CI 1.5–28) and female sex (OR 1.8, 95% CI 1.1–2.9) were associated with a higher risk of long COVID symptoms. Comparison between symptomatic patients infected in the period March–December 2020 (prevalent circulation of wild-type SARS-CoV-2) with those infected in the period January–April 2021 (prevalent circulation of B.1.1.7 Alpha variant) showed a significant modification in the pattern of symptoms belonging to the neurological and cognitive/emotional categories. Our findings confirmed shortness of breath and chronic fatigue as the most frequent long COVID manifestations, while female sex and severe COVID-19 course were the main risk factors for developing lingering symptoms. SARS-CoV-2 variants may induce different long COVID phenotypes, possibly due to changes in cell tropism and differences in viral–host interaction.

## 1. Introduction

Over two and a half years after appearing on the global scene, severe acute respiratory syndrome coronavirus 2 (SARS-CoV-2), the causative agent of coronavirus disease 2019 (COVID-19), still represents a major public health concern, having passed a half billion cases of infection worldwide [1]. While mitigation strategy, vaccines and novel therapeutic options have significantly reduced COVID-19 mortality and severity, chronic symptoms following the acute phase of the disease remain an unresolved issue. The total morbidity resulting from post-COVID-19 consequences has been estimated to be comparable to, or even greater than, the burden from the acute infection [2].

Acute COVID-19 can span from asymptomatic to life-threatening and fatal disease, with a wide range of symptoms reflecting the multi-organ tropism of SARS-CoV-2 [3]. A subset of patients who recover from the acute disease develops long-lasting sequelae, commonly referred to as “long COVID” or “post-COVID-19” syndrome. In fact, a unifying case definition is still lacking, and the term “long COVID” is used to identify the persistence of a heterogeneous range of symptoms several weeks or months following the infection with SARS-CoV-2 [4]. The National Institute for Health and Care Excellence has distinguished between “ongoing symptomatic COVID-19” when signs and symptoms of COVID-19 are present from 4 to 12 weeks after infection and “post-COVID-19 syndrome” when signs and symptoms persist for more than 12 weeks [5]. The US Centers for Disease Control and Prevention uses the term ‘post-COVID-19 conditions’ when health consequences are present ≥4 weeks after infection [6]. Others have defined “post-acute COVID-19” as symptom persistence beyond 3 weeks, and “chronic COVID-19” referring to symptoms beyond 12 weeks [7].

Clinical presentation of long COVID includes fatigue, dyspnoea, anosmia and dysgeusia, palpitation, impaired memory and concentration, insomnia and a variety of neuropsychiatric disorders as the major manifestations, with several organ systems involved [8,9,10]. Data on chronic sequelae of COVID-19 are continually being updated in the surveys launched by the Office for National Statistics in the UK [11]. Symptomatology can last for long time, as demonstrated in longitudinal, prospective cohort studies of post-discharge COVID-19 patients, showing a substantial burden of symptoms not attributable to alternative diagnoses at 12 months [12,13].

Despite an increasing body of literature, several aspects of long COVID remain poorly understood. Suggested underlying pathophysiological mechanisms include putative viral reservoirs, induced autoimmunity and inflammatory-mediated damage [9,10]. Risk factors and role of biomarkers, as well as the potential impact on long COVID of the vaccination status are still open issues [8,9,10]. Moreover, no information is available so far on potential changes in the long COVID pattern associated with the different viral variants that have emerged since the fall of 2020.

In the present study, we investigated the characteristics of post-COVID-19 patients evaluated 4 to 12 weeks after being discharged from Careggi University Hospital, Florence, Italy, during a 12-month period (June 2020–June 2021) within a follow-up program for post-acute COVID-19 patients.

## 2. Materials and Methods

We prospectively enrolled patients admitted to the post-COVID-19 outpatient service at the Infectious and Tropical Diseases Unit, Careggi University Hospital, Florence, Italy, 4–12 weeks after hospital discharge, between June 2020 and June 2021. The period reflects the first 12 months of activity of the service, which started at the end of May 2020, soon after the end of the so-called ‘first wave’ of the pandemic, and was supported by a dedicated program of the Tuscany Region [14].

The program was based on a multidisciplinary approach, with the contribution of infectious disease specialists, pulmonologists, cardiologists, immunologists and physiotherapists.

All patients discharged from the hospital were offered a clinical visit. Exclusion criteria were (i) patients discharged for more than 12 weeks; (ii) patients unable to attend the visit because of hospitalization or residents in care facilities; (iii) patient refusal.

Data on previous hospital admissions were retrieved from electronic medical records. Disease severity was classified as mild, moderate, severe and critical, according to World Health Organization (WHO) definitions [15]. A detailed post-discharge clinical history was collected through a standardized questionnaire focused on persistence of symptoms potentially related to recent SARS-CoV-2 infection. Symptom count included any self-reported symptom persisting at the time of the follow-up visit. Post-discharge symptoms resolved before the visit were not considered in the count. A full physical examination was performed.

Descriptive analysis was used to illustrate population characteristics. Categorical variables were evaluated with the Pearson chi-square/Fisher exact test, as appropriate. Continuous variables were evaluated with the Mann–Whitney test. A multivariate logistic regression by forward stepwise approach was performed, aiming to investigate the statistical association between symptom persistence and demographic and clinical features, including sex, age, comorbidities, COVID-19 severity and ICU admission and COVID-19 therapies and oxygen support required. Age- and sex-adjusted logistic regression was used to compare symptom frequencies between different periods of observation.

## 3. Results

### 3.1. Baseline Features

Overall, 428 patients were enrolled, including 174 women (41%), with a median age of 64 years (IQR 54–76, range 20–93). Most common comorbidities included arterial hypertension (185, 43%), coronary heart disease (CHD) (83, 19%), diabetes (83, 19%) and obesity (53, 12%). Only two patients (0.5%) were vaccinated against SARS-CoV-2 before hospitalization for COVID-19. Regarding COVID-19 severity, 178 (41.6%) patients experienced severe to critical disease and 64 (15%) required admission to the intensive care unit. Ninety-two (22%) were treated with remdesivir, 297 (69%) received corticosteroids, and 48 (11%) received tocilizumab or other immune modulators. Data on oxygen support registered low flow supplementation in 234 patients (55%), high flow nasal cannula (HFNC) in 33 (8%), non-invasive ventilation (NIV) in 100 (23%) and mechanical ventilation (MV) or extra corporeal membrane oxygenation (ECMO) in 21 (5%). Demographic and clinical data are reported in detail in Table 1.

### 3.2. Long COVID Features

**Figure 1:**
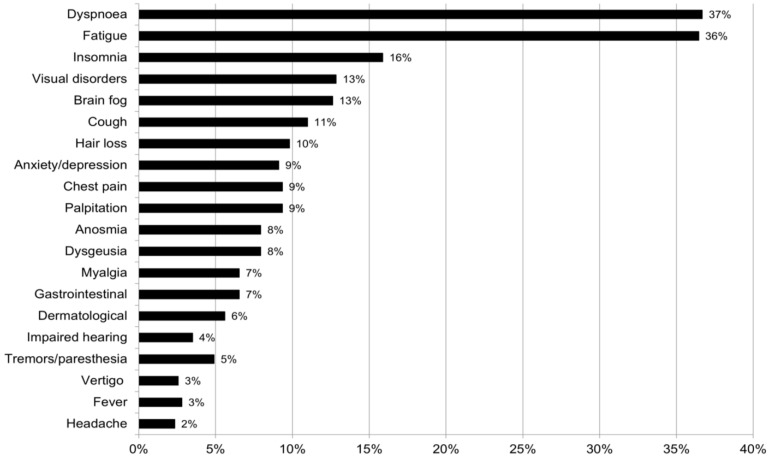
Prevalence of lingering symptoms in the study population (N = 428).

The follow-up visit was performed a median 53 days (IQR 40–64) after hospital discharge and 69 days (IQR 55–82) after the first positive result by PCR on nasopharyngeal swab. Overall, 325 patients (76%) reported at least one persistent symptom, while 154 (36%) and 92 (21%) described more than two or three persistent symptoms, respectively. The most frequent symptoms were shortness of breath (157; 37%), chronic fatigue (156; 36%), insomnia (68; 16%), visual disorders (55; 13%), brain fog (54; 13%) and cough (47; 11%) (Figure 1).

### 3.3. Risk Factors

Persistence of one or more symptoms was significantly more prevalent in women (81% vs. 72% in men), as well as those requiring ICU stay (87.5% vs. 74% in non-ICU patients) and in patients treated with tocilizumab or other immunosuppressant (97% vs. 74% in those non-treated). Moreover, a statistical association with length of hospital stay, oxygen support requirement and COVID-19 severity was observed (Table 2). A lower prevalence of prolonged symptoms was associated with diabetes (29% vs. 16% in non-diabetic patients).

By multivariable analysis, advanced oxygen supplementation (HFNC or higher support) (OR 1.9, 95% CI 1.1–3.3), use of immunosuppressant drugs (OR 6.6, 95% CI 1.5–28.5) and female sex (OR 1.8, 95% CI 1.1–3.0) were independently associated with a higher risk of developing long COVID symptoms, while diabetes (OR 0.4, 95% CI 0.3–0.8) was inversely associated with symptom persistence (Table 2).

### 3.4. Temporal Trend of Persistent Symptoms

In order to investigate a potential change in long COVID pattern over time, we compared data from patients who acquired the infection between March and December 2020, corresponding to the period of prevalent circulation of the original Wuhan SARS-CoV-2 strain in Italy, with those from patients infected in January–April 2021 (prevalent circulation of B.1.1.7 Alpha variant) [16]. The groups presented no significant difference by age, sex, comorbidities, COVID-19 therapies and COVID-19 severity (data not shown). Adjusting the population by sex and age, we observed that the prevalence of patients reporting persistent symptoms was similar (78% vs. 72% in 2020 and 2021, respectively), as well as the frequency of the most common chronic symptoms, namely shortness of breath (33% vs. 42%) and chronic fatigue (38% and 35%). Nevertheless, significant differences were observed in the frequencies of some symptoms: in the latter period, the prevalence of myalgia (10% vs. 4%; OR 2.5, 95% CI 1.2–5.7), brain fog (16% vs. 10%; OR 1.8, 95% CI 1.1–3.3) and anxiety/depression (13% vs. 6%; 2.4, 95% CI 1.2–4.7) significantly increased, while anosmia and dysgeusia were less frequent (2% vs. 12%; 0.4, 95% CI 0.2–0.9 and 4% vs. 11%, 0.2, 95% CI 0.1–0.5, respectively) (Table 3).

## 4. Discussion

Long COVID is a complex, heterogeneous condition with multisystem involvement, which represents an emerging healthcare challenge. The first case series about persistent symptoms after acute COVID-19 was published in July 2020 and described a high rate of sequelae among 143 Italian patients a mean of 60 days after COVID-19 hospitalization [17]. Before SARS-CoV-2 emergence, several pathogenic organisms, including bacteria, viruses and parasites, have been associated with post-acute infection syndromes (PAIS), whose underlying mechanisms have been poorly investigated so far [18]. Long COVID shares many features with chronic illnesses triggered by other infectious agents, especially when it develops after non-severe COVID-19, suggesting the involvement of common etiopathogenetic mechanisms. Notably, the epidemic of SARS in 2002–2004 led to an estimated prevalence of post-acute sequelae around 10–20% within a follow-up period ranging from 2 months to 12 years, the most frequent symptoms being fatigue and neuropsychiatric complaints such as sleep disturbances, irritability, depression, anxiety and memory impairment [19].

In our cohort, chronic fatigue and shortness of breath were the most prevalent symptoms reported by more than one in three participants. COVID-19 is primarily a respiratory infection, characterized by interstitial lung infiltrates and vascular disease, thereby it is not surprising that long-term pulmonary consequences are a focal point of the long COVID syndrome. Both radiological and physiological abnormalities of the lungs have been documented several months after recovering from the acute illness. Impaired diffusion capacity for carbon monoxide (DLCO, a marker of pulmonary vascular integrity) was estimated to occur in 39% of cases in a systematic review, being twice as frequent in severe than in non-severe cases [20]. A small prospective study from Wuhan, following up 83 survivors of severe COVID-19 pneumonia at 3-month intervals up to 12 months after hospital discharge, showed that although pulmonary function tests improved over time, DLCO remained <80% of predicted in 33% patients, and radiological abnormalities such as ground-glass opacities were still present in 24% patients at 12 months [21].

Long COVID fatigue may overlap with many aspects of myalgic encephalomyelitis/chronic fatigue syndrome (ME/CFS), which is a core manifestation of several PAIS, both having long-term symptom durations, impaired daily activity, exertional intolerance and post-exertional malaise, along with neurocognitive and sensory disorders [22]. Overall, it has been postulated that several factors and mechanisms play a role in the development of ME/CFS, including chronic inflammation damage at the central and peripheral level, as might a number of psychological and social factors, and the same scenario may be assumed for post-COVID-19 fatigue [9].

Conditions that are widely acknowledged as predictors for poor outcome during the acute illness, such as male sex, increasing age and chronic comorbidities, are likely to be less important in the pathophysiology of long COVID [23]. In our cohort, female sex was the only pre-existing feature associated with a greater risk of long COVID symptoms. Middle age in women has been identified as an at-risk profile of experiencing post-acute COVID consequences in several studies [12,23,24,25,26]. Mechanisms are likely multifactorial, ranging from biology and socioeconomic condition to the wider determinants of health [27]. A possible explanation underpinning sex biases in COVID-19 may include a difference in immune response against SARS-CoV-2 due to genetic and hormonal factors. Female patients showed more robust T-cell activation, faster antibody responses and lower plasma levels of inflammatory innate immune cytokines than male patients during acute SARS-CoV-2 infection [28,29]. A stronger immune response in women than in men may represent a double-edged sword, reducing the risk of severe outcome of acute COVID-19 but leading to a range of debilitating ongoing symptoms weeks to months after the acute disease [30]. A key role of sex hormones is supported by the absence of sex differences in the long COVID pattern among paediatric patients [31].

Other variables found to be independently associated with lingering symptoms were the need for immunosuppressant drugs and advanced oxygen support during the hospital admission, both proxy for more severe COVID-19 course. Correlation between COVID-19 severity and risk of developing chronic consequences have been previously reported in large cohort studies [12,21,25]. However, this association is robust for respiratory sequelae, while it may be less evident when considering chronic fatigue [24,32].

To the best of our knowledge, the association between long COVID patterns and different viral variants has not been previously assessed. In this regard, we provided novel information by comparing long COVID symptoms of patients who acquired COVID-19 during the period of circulation of the Wuhan original strain (March 2020–December 2020) with those of patients infected when the B.1.1.7 Alpha variant was dominant in Italy (January–June 2021). The B.1.1.7 or Alpha SARS-CoV-2 variant, isolated for the first time in the UK in September 2020, was associated with an increased transmissibility and more severe illness compared with the original Wuhan strain, fulfilling the definition of variant of concern (VOC) [33]. Although the two subgroups were similar by demographic and clinical features, significant differences in the pattern of long COVID sequelae were observed, especially among symptoms belonging to the neurological and cognitive–emotional sphere. For instance, in the latter period, a sharp reduction in reporting of chemosensory dysfunction was found. A similar discrepancy also characterized the clinical presentation of acute infection with the B.1.1.7 Alpha variant [34]. Less severe olfactory dysfunction was also reported during acute infection with the Delta (B.1.617.2) and the Omicron (B.1.1.529) variants with respect to wild-type SARS-CoV-2, likely due to changes in cell tropism and interaction with proteins that promote virus uptake (ACE-2, TMPRSS2, and TMEM16F) induced by variant-specific Spike protein mutation [35,36,37,38]. On the contrary, the increased prevalence of neurocognitive symptoms such as brain fog, myalgia and psychiatric disorders observed after January 2021 may reflect not only a different viral–host interaction but also the cumulative burden of negative psychological and social factors associated with the prolonged COVID-19 pandemic. Survivors of COVID-19 have been identified to be at increased risk of psychiatric sequelae, especially anxiety disorders and post-traumatic stress disorder-like picture, which can potentially evolve into brain fog and other neurocognitive impairments [39].

Finally, whether the SARS-CoV-2 vaccine can reduce the chance of developing long COVID symptoms and/or can modify long COVID course is still debated. In our study, the enrolment ended in the very early phase of the Italian SARS-CoV-2 vaccine campaign, and only two patients with a history of vaccination before acquiring COVID-19 were included, thus hindering any possible inference. A rapid evidence review carried out by the UK Health Security Agency (UKHSA) concluded that people who received two doses of a vaccine against COVID-19 halved the risk of developing long COVID symptoms compared with those unvaccinated [40]. More recently, a study based on the US Department of Veterans Affairs national healthcare databases suggested that vaccines offer less protection than expected against lingering symptoms, by only about 15%, thus making it not possible to rely on it as a sole mitigation strategy for the reduction in long-term consequences of SARS-CoV-2 infection [41]. On the other hand, a significant decrease in the odds of ongoing symptoms was observed after COVID-19 vaccination in a large UK cohort of patients who were experiencing long COVID, with a sustained improvement as high as 8.8% after a second dose over the next nine weeks [42].

The burden of long COVID is set to grow, according to the increasing number of infections and re-infections with SARS-CoV-2 worldwide. Further studies and novel approaches are warranted to better characterise this syndrome. First of all, it is essential to achieve a consensus upon the nomenclature and definition to assess its incidence, subtypes and severity [43]. Moreover, a core outcome set approach, defined as “an agreed standardised collection of outcomes which should be measured and reported, as a minimum, in all trials for a specific clinical area” has been proposed to overcome the substantial heterogeneity that exists in the outcomes that are reported in post-COVID-19 studies [44]. A greater consistency of definitions and outcomes will allow better comparisons and synthesis of research data, possibly contributing to improve insights into this syndrome and ultimately to develop effective therapeutic measures for these patients.

## Figures and Tables

**Table 1:** Demographic and clinical details of the cohort of 428 patients evaluated at the Careggi University Hospital (Florence, Italy) 4–12 weeks after hospitalization for COVID-19, between June 2020 and June 2021.

**Sex**	***n***	**%**
Male	254	59%
Female	174	41%
**Age**	**Years**	**IQR**
Median	64	54–76
Range	20–93	
**Comorbidities**	***n***	**%**
Hypertension	185	43%
COPD	17	4%
CHD	83	19%
Diabetes	83	19%
CKD	18	4%
Obesity	53	13%
	**Days**	**IQR**
Hospital stay	10	6.5–18
Time discharge to visit	53	40–64
Time diagnosis to visit	68.5	55–82
**ICU admission**	***n***	**%**
	64	15%
**COVID-19 therapies**	***n***	**%**
Remdesivir	92	22%
Antiretrovirals (LPV/r, DRV/c)	76	18%
Hydroxychloroquine	88	21%
Steroids	297	69%
Tocilizumab	35	8%
Ruxolitinib	13	3%
Convalescent plasma	34	8%
Monoclonal antibodies	1	0.2%
**Oxygen Support**	***n***	**%**
None	40	9%
Low flow	234	55%
HFNC	33	8%
NIV	100	23%
MV	20	5%
ECMO	1	0.2%
**WHO severity scale** [15]	***n***	**%**
Asymptomatic	25	6%
Mild	62	14%
Moderate	163	38%
Severe	97	23%
Critical	81	19%

**Table 2:** Risk factors for long COVID persistent symptoms in a cohort of 428 patients evaluated at the Careggi University Hospital (Florence, Italy) 4–12 weeks after hospitalization for COVID-19, between June 2020 and June 2021.

Variable	Persistent Symptoms		Univariable	Multivariable
	No (N = 103)	Yes (N = 325)	*p*-Value	OR (95% CI)
**Sex *n* (%)**				
M	70 (28)	184 (72)		
F	33 (19)	141 (81)	0.041	1.8 (1.1–3.0)
**Median age (years, IQR)**	62 (52–76)	64 (54–76)	0.551	
Hospital stay (median days, IQR) Time diagnosis to visit (days) Time discharge to visit (days)	8 (5–13)	11 (7–20)	<0.001	
	64 (51–81)	69 (56–83)	0.051	
	52 (37–65)	53 (40–64)	0.773	
**ICU admission**				
No	95 (26)	269 (74)		
Yes	8 (12.5)	56 (87.5)	0.019	
**Comorbidities**				
Arterial hypertension	46 (25)	139 (75)	0.736	
COPD	2 (12)	15 (88)	0.383	
CHD	26 (31)	57 (69)	0.085	
Diabetes	30 (36)	53 (64)	0.004	0.4 (0.3–0.8)
CKD	4 (22)	14 (78)	0.852	
Obesity	10 (19)	43 (81)	0.301	
**COVID-19 therapies**				
Remdesivir	26 (28)	66 (72)	0.288	
Steroids	78 (26)	219 (74)	0.109	
Immunosuppressant drugs	1 (3)	34 (97)	0.001	6.6 (1.5–28.5)
Convalescent plasma	11 (32)	23 (68)	0.239	
Advanced oxygen support *				
No	78 (28)	196 (71)		
Yes	25 (16)	129 (84)	0.004	1.9 (1.1–3.3)
**WHO severity scale**				
Asymptomatic	10 (40)	15 (60)		
Mild	12 (19)	50 (81)		
Moderate	42 (26)	121 (74)		
Severe	33 (34)	64 (66)		
Critical	6 (7)	75 (93)	<0.001	

* defined as high flow nasal canulae (HFNC) or higher support.

**Table 3:** Comparison of symptom frequencies in March–December 2020, corresponding to the period of prevalent circulation of original Wuhan SARS-CoV-2 strain in Italy, with those from patients infected in the period January–April 2021 (prevalent circulation of B.1.1.7 Alpha variant).

	Total *n* (%) N = 428	March–December 2020 *n* (%) N = 245	January–June 2021 *n* (%) N = 183	OR (95% CI) ^1^	*p*-Value
Total symptoms	325 (76)	192 (78)	133 (72)	0.7 (0.5–1.2)	0.182
fatigue	156 (37)	92 (38)	64 (35)	0.9 (0.6–1.3)	0.594
fever	12 (3)	6 (2)	6 (3)	1.3 (0.4–4.1)	0.656
shortness of breath	157 (37)	81 (33)	76 (42)	1.4 (0.9–2.1)	0.071
palpitation	40 (9)	22 (9)	18 (10)	1.1 (0.6–2.1)	0.782
cough	47 (11)	28 (11)	19 (10)	0.9 (0.5–1.7)	0.731
chest pain	40 (9)	21 (9)	19 (11)	1.2 (0.6–2.3)	0.589
insomnia	68 (16)	41 (17)	27 (15)	0.8 (0.5–1.4)	0.535
headache	10 (2)	5 (2)	5 (3)	1.3 (0.4–4.6)	0.693
**brain fog**	**54 (13)**	**24 (10)**	**30 (16)**	**1.8 (1.1–3.3)**	**0.039**
**dysgeusia**	**34 (8)**	**26 (11)**	**8 (4)**	**0.4 (0.2–0.9)**	**0.025**
**anosmia**	**34 (8)**	**30 (12)**	**4 (2)**	**0.2 (0.1–0.5)**	**0.001**
gastrointestinal	28 (7)	15 (6)	13 (7)	1.2 (0.5–2.6)	0.663
visual disorders	55 (13)	32 (13)	23 (13)	0.9 (0.5–1.7)	0.830
**myalgia**	**28 (7)**	**10 (4)**	**18 (10)**	**2.5 (1.2–5.7)**	**0.021**
hair loss	42 (10)	24 (10)	18 (10)	1.0 (0.5–2.0)	0.961
vertigo	11 (3)	7 (3)	4 (2)	0.8 (0.2–2.7)	0.671
impaired hearing	15 (4)	12 (5)	3 (2)	0.3 (0.1–1.2)	0.098
tremors/paraesthesia	21(5)	13 (5)	8 (4)	0.8 (0.3–2.0)	0.618
**anxiety/depression**	**39 (9)**	**15 (6)**	**24 (13)**	**2.4 (1.2–4.7)**	**0.013**
dermatological	24 (6)	14 (6)	10 (5)	1.0 (0.4–2.2)	0.941

^1^ Age- and sex-adjusted analysis. In bold significant association.

## Data Availability

Most data are available in the text and in the table. Further data will be provided on request to the corresponding author.
